# Effects of a 6-month Yoga Intervention on Cognition & Anterior Cingulate Cortex: Results from the SAY Exercise Trial

**DOI:** 10.1093/geroni/igaf122.268

**Published:** 2025-12-31

**Authors:** Neha Gothe, Angeles Tepper, Paul Camacho, Stephanie Voss, Jessica Hayes, Brad Sutton, Jessica Damoiseaux, Edward McAuley

**Affiliations:** Northeastern University, Boston, Massachusetts, United States; Northeastern University, Boston, Massachusetts, United States; University of Illinois Urbana-Champaign, Urbana, Illinois, United States; Mass General Brigham, Boston, Massachusetts, United States; Wayne State University, Detroit, Michigan, United States; University of Illinois Urbana-Champaign, Urbana, Illinois, United States; Wayne State University, Detroit, Michigan, United States; University of Illinois Urbana-Champaign, Urbana, Illinois, United States

## Abstract

Lifestyle behaviors such as exercise continue to demonstrate neuroprotective effects with aging. A growing body of evidence suggests the role of aerobic exercise; however, little is known about alternative exercise approaches such as yoga. Sedentary older adults (N = 145, Mean age = 64.2) were randomized to one of three exercise groups: Hatha yoga, aerobic exercise, stretching-toning. Participants engaged in group exercise classes 3x/week, 60mins/session for 6 months. Neurocognitive measures including the Flanker inhibition task and Sternberg working memory task were administered pre and post intervention along with a structural MRI. Linear mixed models demonstrated improved reaction time (RT) on the Flanker incongruent condition for both yoga and aerobic groups (b_yoga = -51.17, p < .001; b_aerobics = -30.38, p = .011) but no significant improvement in the stretching-toning group (b_stretch = -9.69, p = .397). RT on the Sternberg 6-digit condition was also significantly faster among yoga participants (b_yoga = -92.79, p < 0.001; b_aerobics = -6.14, p = 0.805; b_stretch = -25.39, p = 0.317) and this change significantly correlated with changes in anterior cingulate cortex (r =-.41, p=.015) only in the yoga group. There appears to be specificity in the effects observed with the aerobic group demonstrating improvements in inhibitory control whereas yoga demonstrating improvements in both inhibitory control and working memory. For yoga, faster RT on the Sternberg 6-digit condition also associated with increased volume in the anterior cingulate cortex, a brain structure known to be impacted by aerobic exercise and play critical role in executive functioning.

